# Combination of Coenzyme Q_10_ Intake and Moderate Physical Activity Counteracts Mitochondrial Dysfunctions in a SAMP8 Mouse Model

**DOI:** 10.1155/2018/8936251

**Published:** 2018-10-24

**Authors:** C. Andreani, C. Bartolacci, M. Guescini, M. Battistelli, V. Stocchi, F. Orlando, M. Provinciali, A. Amici, C. Marchini, L. Tiano, P. Orlando, S. Silvestri

**Affiliations:** ^1^University of Camerino, via Gentile III da Varano, 62032 Camerino, Italy; ^2^University of Urbino, via Aurelio Saffi, 61029 Urbino, Italy; ^3^Experimental Animal Models for Aging Unit Scientific Technological Area, IRCCS INRCA, via del Fossatello, 60127 Ancona, Italy; ^4^Advanced Technological Center for Aging Research Scientific Technological Area, IRCCS INRCA, via Birarelli 8, 60121 Ancona, Italy; ^5^Polytechnic University of Marche, Department of Life and Environmental Sciences (DISVA), via Brecce Bianche, Ancona, Italy; ^6^Biomedfood srl, Spinoff of Polytechnic University of Marche, via Brecce Bianche, 60131 Ancona, Italy

## Abstract

Aging skeletal muscles are characterized by a progressive decline in muscle mass and muscular strength. Such muscular dysfunctions are usually associated with structural and functional alterations of skeletal muscle mitochondria. The senescence-accelerated mouse-prone 8 (SAMP8) model, characterized by premature aging and high degree of oxidative stress, was used to investigate whether a combined intervention with mild physical exercise and ubiquinol supplementation was able to improve mitochondrial function and preserve skeletal muscle health during aging. 5-month-old SAMP8 mice, in a presarcopenia phase, have been randomly divided into 4 groups (*n* = 10): untreated controls and mice treated for two months with either physical exercise (0.5 km/h, on a 5% inclination, for 30 min, 5/7 days per week), ubiquinol 10 (500 mg/kg/day), or a combination of exercise and ubiquinol. Two months of physical exercise significantly increased mitochondrial damage in the muscles of exercised mice when compared to controls. On the contrary, ubiquinol and physical exercise combination significantly improved the overall status of the skeletal muscle, preserving mitochondrial ultrastructure and limiting mitochondrial depolarization induced by physical exercise alone. Accordingly, combination treatment while promoting mitochondrial biogenesis lowered autophagy and caspase 3-dependent apoptosis. In conclusion, the present study shows that ubiquinol supplementation counteracts the deleterious effects of physical exercise-derived ROS improving mitochondrial functionality in an oxidative stress model, such as SAMP8 in the presarcopenia phase.

## 1. Introduction

Aging is characterized by a progressive decline in skeletal muscle mass and muscular strength [1–3]. In healthy people, there is a 1% per year decline in muscle mass between 20 and 30 years of age. This decline is accelerated above 50 years of age [4, 5]. The progressive decline in muscle mass and strength with aging is known as sarcopenia [1, 6–8]. Sarcopenia is defined as a geriatric syndrome characterized by age-related muscular loss and dysfunction that cause physical disability, a poor quality of life, and death. The prevalence of this pathology in adults under the age of 70 is about 25% but increases up to 40% in 80-year-old or older people [9, 10]. This condition can lead to decreased physical activity increasing the risk of falls in aged individuals [11]. Understanding the mechanisms underneath aging-induced skeletal muscle atrophy and promoting health and mobility in the elderly are crucially important goals in order to develop therapeutic strategies [12]. Several studies pointed towards a critical role of mitochondria and their implication in age-related degenerative processes, and many therapeutic attempts have been focused on mitochondria [13]. Indeed, these organelles play a key role in cellular bioenergetics and represent a sensitive target in muscle cells [14, 15]. Moreover, metabolism of reactive oxygen species (ROS), Ca^2+^ homeostasis, and apoptosis are controlled by mitochondria [16]. Aging of skeletal muscle determines the alteration of the structure and function of these organelles leading to mitochondrial dysfunction [17]. In this context, a growing body of evidence has highlighted a major role of oxidative stress and inflammation in promoting aging of skeletal muscle [18]. Accordingly, it has been recently reported that excessive production of mitochondrial ROS in skeletal muscle is strongly associated with sarcopenia and the impairment of energy homeostasis [19]. In fact, the physiologic equilibrium between ROS production and antioxidant defense is disrupted in aging subjects, and the accumulation of ROS during mitochondrial respiration can cause mutations in mitochondrial DNA (mtDNA) [20] which in turn lead, through a vicious cycle, to further impaired mitochondrial functionality. Moreover, many studies have reported that a decline in mitochondria content may also account for the loss of skeletal muscle mass [18], further impairing oxidative phosphorylation and ATP production [21, 22]. Mitochondrial biogenesis is regulated by the expression of nuclear and mitochondrial genes, controlled by the transcriptional coactivator peroxisome proliferator gamma coactivator-1*α* (PGC-1*α*) [23]. Vainshtein et al. [24] suggested a role of this coactivator also in the regulation of autophagy and mitophagy in skeletal muscle. These two are distinct but interconnected degradation processes aimed at eliminating damaged cellular components in response to stress stimuli. Both mitophagy and autophagy are regulated by autophagy-related genes (Atgs) including Beclin-1 and LC3 [25].

During aging, skeletal muscle fibers gradually lose the capability to remove dysfunctional mitochondria [13]. This condition could further impair mitochondrial respiration and enhance ROS production [26] contributing to the onset of sarcopenia. Previous studies suggested that an appropriate physical activity regimen can counterbalance age-associated muscular deficits by promoting mitochondrial biogenesis [27–29]. Exercise training has been reported to modulate skeletal muscle metabolism, regulating intracellular signaling pathways and thus mediating mitochondrial homeostasis [30, 31]. However, some authors raised doubts regarding the beneficial role of exercise in the elderly, claiming that physical activity-dependent ROS production could exacerbate oxidative damage inside aged skeletal muscles [32–34]. In this scenario, association of physical activity with antioxidant therapies might be an effective strategy to prevent the adverse effects of exercise in the elderly. Coenzyme Q_10_ represents a valuable candidate for oxidative stress prevention and for supporting muscle functionality [35–39]. Coenzyme Q (CoQ) consists of a quinone head which, in mammalian cells, is attached to a chain of 9 (CoQ_9_) or 10 isoprene units CoQ_10_ [40]. In human tissues, the most abundant form is coenzyme Q_10_, while in mice and rats it is coenzyme Q_9_, although CoQ_10_ represents a significant proportion of total CoQ and its level is able to increase following oral supplementation [41–46]. As part of the mitochondrial electron transport chain (ETC), CoQ actively participates in oxidative phosphorylation and plays a key role in energy and redox state balance [47]. In addition, CoQ has been found in other subcellular localizations and in circulating plasma lipoproteins, where it acts as an endogenous lipophilic antioxidant in synergism with vitamin E [48]. Endogenous CoQ_10_ synthesis, the principal source of CoQ [49], has been shown to significantly decrease during aging and in certain degenerative diseases [50, 51], thus triggering cellular dysfunctionality. These evidences underlie the rationale for CoQ use in clinical practice and as a food supplement. CoQ exists in three states of oxidation: ubiquinone (CoQ), the fully oxidized form; ubisemiquinone (CoQH^.^), the partially reduced form; and ubiquinol (CoQH_2_), the fully reduced form. In particular, the CoQH_2_ form has several advantages being more bioavailable and readily usable by the organism not requiring reductive steps [52]. This is of particular relevance in conditions when reductive systems might be less efficient such as during aging or following intense physical exercise. Here, we investigated the effect of a combined approach of mild physical exercise and ubiquinol supplementation on the senescence-accelerated mouse-prone 8 (SAMP8) model in a presarcopenia phase [53, 54]. SAMP strains derived from AKR/J series [55] show senescence acceleration and age-related pathological phenotypes, similar to aging disorders seen in humans. In particular, we focused on SAMP8 mice since they exhibit the most striking features among SAMP strains in terms of life span, fast aging progression due to high oxidative stress status [56, 57], dramatic decrease in muscle mass and contractility [58, 59], and a huge reduction in type II muscle fiber size [60, 61]. The aim of this study is to develop prevention strategies able to preserve skeletal muscle health during aging by maintaining mitochondrial function through regular physical exercise and antioxidant supplementation using a senescence-accelerated mouse-prone model (SAMP8).

## 2. Materials and Methods

### 2.1. SAMP8 Housing and Treatment

**Figure 1:**
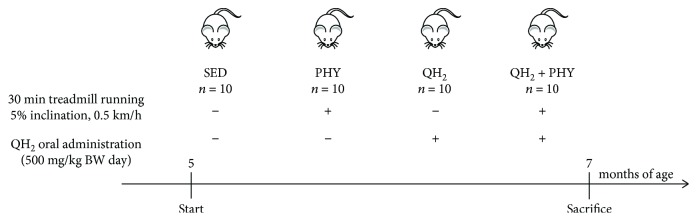
Scheme of SAMP8 mouse study.

Senescence-accelerated mice (SAMP8, Harlan) [58, 59, 62], aged 5 months, have been randomly divided into 4 groups (*n* = 10) as summarized hereafter: untreated controls (SED), trained (PHY), ubiquinol 10-administered (QH_2_), and both trained and ubiquinol 10-supplemented (QH_2_ + PHY). The PHY and QH_2_ + PHY groups underwent treadmill running at 0.5 km/h, on a 5% inclination, for 30 min, 5 days per week, for 2 months up to 7 months of age [63, 64] (Figure 1). The QH_2_ and QH_2_ + PHY groups were supplemented with ubiquinol 10 (Kaneka) (500 mg/kg body weight/day in sunflower seed oil) via oral administration. Such QH_2_ formulation was previously prepared and stored at −80°C in 500 *μ*L aliquots to avoid repeated freezing-thawing cycles. An aliquot was thawed daily in a water bath at 60°C in the dark just prior to administration. An equal amount of sunflower seed oil was given to SED and PHY mouse groups. The animals were bred and housed under controlled temperature (20°C) and a circadian cycle (12-hour light/12-hour dark). The animals were fed on chow diet and water *ad libitum.* Male mice were used for all experiments. The animal procedures followed the 2010/63/EU directive on the protection of animals used for scientific purposes and were approved by the Ethic Committee on Animal Use of the University of Camerino (protocol number 14/2012).

### 2.2. Tissue Collection and Analysis

Mice were rapidly sacrificed by isoflurane inhalation followed by cervical dislocation, two days after the last exercise/administration session, to avoid possible metabolic effects of the last exercise/administration bout. *Gastrocnemius* (GA), *tibialis anterior* (TA), *soleus* (SO) muscle, and cardiac muscle were carefully excised. GA samples were either immediately used for flow cytometry (FACS) analysis or preserved in liquid nitrogen for mtDNA quantification or mRNA extraction. TA and cardiac muscles were used for CoQ_9_ and CoQ_10_ (total and oxidized form) quantification. TA muscles were used also for protein extraction and Western blot analysis, while SO samples were fixed in 3% glutaraldehyde for 4 hours, to be analyzed for fiber morphology, number, and ultrastructure of mitochondria by electron microscopy. Other tissues including liver, spleen, and kidneys were recovered for eventual future applications and preserved at −80°C.

### 2.3. Coenzyme Q_9_ and Q_10_ Extraction and Quantification

TA and cardiac muscles were mechanically homogenized (two bouts at 30 Hz for 5 min) in propanol (Sigma) using 7 mm steel beads (Qiagen) and TissueLyser II (Qiagen). After centrifugation (2 min at 20,000 g, 4°C), 40 *μ*L of the supernatant was injected into a high-performance liquid chromatography (HPLC) apparatus with an electrochemical detector (ECD), model 3016 by Shiseido Co. Ltd, to measure total coenzymes Q_9_ and Q_10_ and Q_9_ oxidative status. The mobile phase was 50 mM sodium perchlorate in methanol/distilled water (95/5 *v*/*v*) with a flow rate of 0.2 mL/min. Using a column-switching system, coenzymes were eluted from the concentrating column by mobile phase 2, 50 mM sodium perchlorate in methanol/isopropanol (70/30 *v*/*v*) with a flow rate of 0.08 mL/min. The column oven was set at 40°C. Pumps one and two of model 3001, autosampler model 3033, and switch valve model 3012; concentration column CQC (C8 DD; 10 mm × 4.0 mm ID); and separation column CQS (C18 AQ; 150 mm × 2.0 mm ID, particle size at 3 *μ*m diameter) were used, all from Shiseido Co. Ltd. A peculiarity of the system was the use of a postseparation reduction column (Shiseido CQR) capable of fully reducing the peak of ubiquinone. CoQ_9_ and CoQ_10_ standard solutions were previously prepared in ethanol and stored at −80°C. The oxidation potential for ECD was 650 mV. TA and cardiac muscle contents of CoQ_9_ and CoQ_10_ were expressed as *μ*g/g muscle and the oxidized form as percentage of total CoQ_9_.

### 2.4. Electron Microscope Analysis

Control and treated SO samples were washed and immediately fixed with 2.5% glutaraldehyde in 0.1 M phosphate buffer for 1 hour, postfixed with 1% of osmium tetroxide (OsO_4_) in the same buffer for 2 hours, and embedded in araldite, as previously reported [65, 66]. The sections were collected on 400-mesh nickel grids, stained with uranyl acetate, lead citrate and finally analyzed with an electron microscope at 80 kV.

### 2.5. Flow Cytometry Analysis

#### 2.5.1. Skeletal Muscle Dissociation

GA muscles were dissociated into single-cell suspensions using a skeletal muscle dissociation kit (Miltenyi Biotec) according to the manufacturer's instructions. Mechanical disaggregation was performed via gentleMACS Dissociator using the m_muscle_01 program (Miltenyi Biotec).

#### 2.5.2. Cell Viability and Cell Count

Cell viability and cell count of the obtained single-cell suspensions were evaluated using Guava ViaCount® Reagent Kit (Millipore) that discriminates among viable, apoptotic, and dead cells. Briefly, the assay exploits a mixture of cell membrane-permeable (red) and cell membrane-impermeable (yellow) DNA-binding fluorescent probes, diluted 1 : 10 in PBS, and used to stain cells immediately before reading. Cells were incubated with reagent for 5 min in the dark, and the analysis of the distribution allows the discrimination of the percentage of cell debris (R−/Y−), live cells (R+/Y−), and dead cells (R+/Y+) with Guava ViaCount software using a Guava easyCyte™ flow cytometer (Millipore).

#### 2.5.3. Mitochondrial Membrane Depolarization

Mitochondrial membrane depolarization was measured by incubating 2.5 × 10^5^ viable muscle cells with MitoProbe™ DiIC1(5) (Life Technologies) (40 nM final concentration) at 37°C for 20 min in the dark. During the experimental setup, a suspension of control cells, before staining, was incubated with 1 *μ*L of carbonyl cyanide 3-chlorophenylhydrazone (CCCP) 50 mM for 5 min at 37°C in the dark. After washing with phosphate-buffered saline (PBS), cells were centrifuged at 300 g for 5 min at room temperature and finally resuspended in PBS and analyzed using the Guava easyCyte™ flow cytometer (Millipore), equipped with a red laser at 633 nm. Using the Guava InCyte software, a gate relative to cells containing depolarized mitochondria was arbitrarily set using as a reference CCCP-treated cells assuming that in this condition 90% of the cells contained depolarized mitochondria. This gate was subsequently used for all further analyses.

### 2.6. Mitochondrial DNA (mtDNA) Quantification

To assess mtDNA content, DNA was extracted from GA muscle using QIAamp DNA Mini kit (Qiagen) and then used for quantitative real-time PCR (qRT-PCR) on the StepOne Plus system (Applied Biosystems). The 36B4 gene was used as a nuclear DNA (nDNA) marker while the COX1 gene was used for mtDNA. The primers used are summarized in Table 1. Briefly, 10 ng of DNA was amplified using 1x SYBR Select Master Mix (Applied Biosystems), using the following protocol: 10 min denaturation at 95°C, followed by 45 cycles (95°C for 15 sec, 60°C for 15 sec, and 72°C for 30 sec) and melting curve (95°C for 15 sec, 60°C for 30 sec, and 95°C for 15 sec). Relative copy number quantification was carried out using the ΔΔCt method.

### 2.7. Western Blot Assay

TA muscle samples were mechanically homogenized in RIPA buffer (0.1% SDS, 1% NP40, and 0.5% CHAPS) supplemented with protease inhibitors aprotinin, sodium orthovanadate, and phenylmethylsulfonyl fluoride (Sigma-Aldrich). Lysates were incubated on ice for 30 min and then centrifuged at 16.000 g, 4°C, for 20 min. The supernatant was collected, quantified via Bradford method (Bio-Rad), and stored in aliquots at −80°C to avoid repeated freezing-thawing cycles. For Western blot analysis, an equal amount of protein lysates (20–40 *μ*g depending on the protein assayed) were separated onto Criterion™ TGX™ precast gels (Bio-Rad) and transferred to a polyvinylidene difluoride (PVDF) membrane (Millipore) using Criterion™ Blotter (Bio-Rad). Membranes were blocked with 5% BSA-TBS-T and then overnight incubated with primary antibodies at 4°C. Secondary antibody binding was performed at RT for 1 hour. After TBS-T washing, immunoreactive bands were incubated with enhanced chemiluminescent reagent (EuroClone) and detected via ChemiDoc™ XRS+ System (Bio-Rad). Densitometry analysis was accomplished through ImageJ software using H2B (nuclear), VDAC1 (mitochondrial), and *β*-actin (total) as protein normalizers. The results are representative of at least three independent experiments. The antibodies used are listed in Table 2.

### 2.8. Gene Expression Analysis

Total RNA was extracted from GA muscles. RNA purification was performed using the E.Z.N.A.® Total RNA Kit I (Omega Bio-tek) according to the manufacturer's instructions, and contaminant DNA was digested with DNase I enzyme (Ambion). cDNA was synthesized using the Maxima Reverse Transcriptase kit (Thermo Fisher Scientific). Real-time PCR amplifications were conducted using SensiFAST SYBR Green (Bioline) according to the manufacturer's instructions, with 300 nM primers and two *μ*L of cDNA (20 *μ*L final reaction volume). Specific primers used are listed in Table 3.

Thermocycling was conducted using LightCycler 480 (Roche) initiated by a 2 min incubation at 95°C, followed by 40 cycles (95°C for 5 sec, 60°C for 5 sec, and 72°C for 10 sec) with a single fluorescent reading taken at the end of each cycle. Each reaction was conducted in triplicate and completed with a melting curve analysis to confirm the specificity of amplification and lack of primer dimers. Quantification was performed according to the ΔCq method, and the expression levels of GAPDH and S16 were used as a reference [67].

## 3. Statistical Analysis

Data are presented as mean ± SEM. All statistical analyses were performed using GraphPad Prism® 6.0 software. Unpaired two-tailed *t*-test was employed when 2 groups were compared and ANOVA for comparison between three or more groups. Two-way ANOVA with Bonferroni correction for multiple comparisons was used when 3 or more groups were compared over time. The GraphPad Prism routine for outlier identification was used to identify any out-of-range values to be excluded from the statistical analysis.

## 4. Results

### 4.1. Combination of Physical Activity and Ubiquinol Supplementation Increases Q_9_ and Q_10_ Content in Cardiac Muscles and Lowers the Oxidation of Endogenous Coenzyme Q_9_

**Figure 2:**
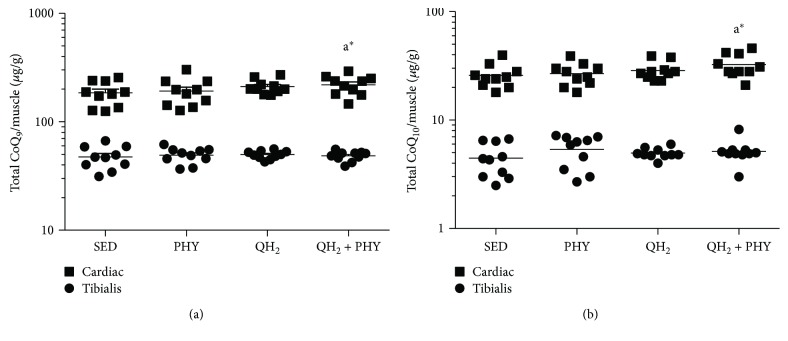
Total coenzyme Q_9_ (a) and Q_10_ (b) levels in cardiac and *tibialis anterior* muscles, expressed as *μ*g coenzyme/g of muscle in sedentary (SED), physical exercise (PHY), ubiquinol (QH_2_), and ubiquinol associated with physical exercise (QH_2_ + PHY) mouse groups (*n* = 10). ^∗^ *p* < 0.05; a = *vs.* SED.

Total coenzyme Q_9_ and Q_10_ (CoQ_9_ and CoQ_10_) levels and oxidative status of coenzyme Q_9_ were quantified by an HPLC-ECD instrument on skeletal and cardiac muscles. The results were normalized on muscle weight and expressed as *μ*g/g of muscle. Coenzyme Q levels were very different between skeletal and cardiac muscles, the latter showing remarkably higher levels of both coenzymes (Figure 2). After ubiquinol and physical exercise (QH_2_ + PHY) treatment, both coenzymes were significantly increased in the cardiac tissue, in particular +23% CoQ_9_ (*p* = 0.05, Figure 2(a)) and +27% CoQ_10_ (*p* = 0.03, Figure 2(b)) with respect to the sedentary group.

**Figure 3:**
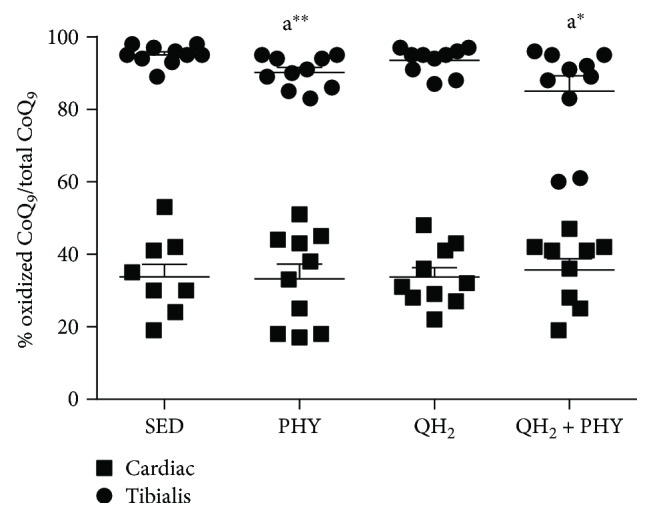
Oxidized coenzyme Q_9_ level in cardiac and *tibialis anterior* muscles, expressed as percentage of oxidized of coenzyme Q_9_ in sedentary (SED), physical exercise (PHY), ubiquinol (QH_2_), and ubiquinol associated with physical exercise (QH_2_ + PHY) mouse groups (*n* = 10). ^∗^ *p* < 0.05 and ^∗∗^ *p* < 0.01; (A) = *vs.* SED.

To evaluate the effect of exogenous CoQ supplementation on the oxidative status of endogenous coenzyme Q_9_, its oxidized form was measured as well. As shown in Figure 3, skeletal muscle is characterized by a higher extent of oxidation (on average 95% of Q_9_ is oxidized) compared to cardiac muscle (35% of oxidized Q_9_). Ubiquinol supplementation alone was not able to lower the oxidation of endogenous muscular CoQ. On the contrary, a significant decrease of oxidized coenzyme Q_9_ was observed in skeletal muscle after regular physical exercise alone or in combination with ubiquinol supplementation (Figure 3, −4.7%, *p* = 0.009, and −3.6%, *p* = 0.03, respectively).

### 4.2. Physical Exercise Alone or in Combination with Ubiquinol Administration Stimulates Muscle Hypertrophy in SAMP8 Mice

**Figure 4:**
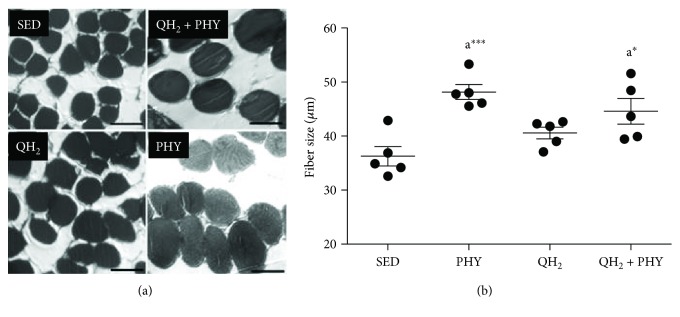
(a) Representative microphotographs of fibers (SED, bar = 25 *μ*m; PHY, bar = 65 *μ*m; QH_2_, bar = 35 *μ*m; and QH_2_ + PHY, bar = 55 *μ*m). (b) Fiber size quantification of *soleus* muscle, expressed in *μ*m, in sedentary (SED), physical exercise (PHY), ubiquinol (QH_2_), and ubiquinol associated with physical exercise (QH_2_ + PHY) mouse groups (*n* = 5). ^∗^ *p* < 0.05 and ^∗∗∗^ *p* < 0.001; (A) = *vs.* SED.

To evaluate the impact of the different treatments on muscle fiber atrophy/hypertrophy, fiber diameter was measured for each experimental condition. Morphometrical analyses of fiber diameter revealed a gradual increase in the ubiquinol (QH_2_), ubiquinol and exercise (QH_2_ + PHY), and exercise (PHY) groups, with the PHY fibers having the largest average diameter (+33% compared to the SED group, *p* = 0.0009, Figures 4(a) and 4(b)). Ubiquinol treatment alone did not produce any significant variation in fiber size, nor was it able to outweigh the effect of physical exercise alone (+23%; *p* = 0.02). These data suggest that physical exercise alone or in combination with ubiquinol is able to induce muscle fiber hypertrophy.

### 4.3. Ubiquinol Supplementation Is Able to Improve Mitochondrial Structure and Morphology Counteracting Physical Activity-Induced Mitochondrial Depolarization

**Figure 5:**
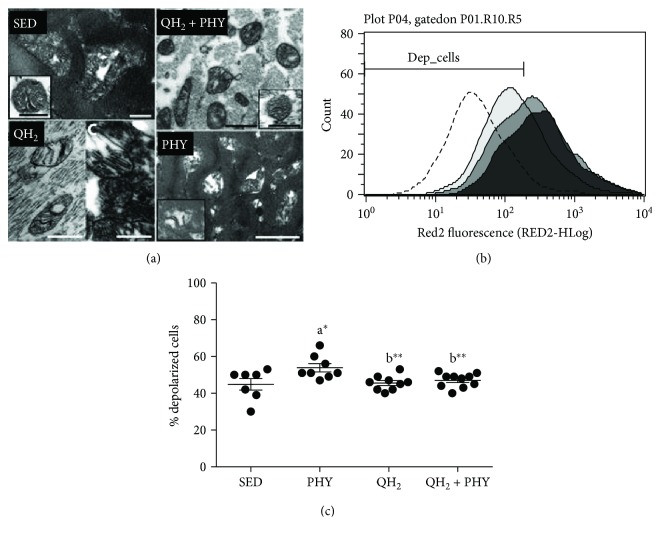
(a) TEM analysis of mitochondrial ultrastructure of *soleus* muscle (SED, bar = 200 nm; inset SED, bar = 500 nm; PHY, bar = 1 *μ*m; QH_2_, bar = 500 nm; and QH_2_ + PHY, inset, and QH_2_ + PHY, bar = 500 nm). (b) Mitochondrial membrane depolarization expressed as Red2 Fluorescent (RED2-HLog). (c) Percentage of depolarized cells of *gastrocnemius* muscle, respectively, in sedentary (black histogram; SED), physical exercise (light grey histogram; PHY), ubiquinol (dark grey histogram; QH_2_), and ubiquinol associated with physical exercise (dark grey histogram; QH_2_ + PHY) mouse groups (*n* = 10). Dashed histogram (b) represents sample control treated with CCCP.^∗^ *p* < 0.05 and ^∗∗^ *p* < 0.01; (A) = *vs.* SED and (B) = *vs.* PHY.

Mitochondrial ultrastructure was evaluated in skeletal muscle by transmission electron microscopy (TEM), and at functional level, mitochondrial membrane potential was evaluated in dissociated skeletal muscle cells by flow cytometry using a Nernstian fluorescent probe. As shown in Figure 5(a), in the SED experimental group, mitochondria appeared rounded or elongated, strongly damaged with rather dilated and disorganized cristae. Strikingly, muscle mitochondria of the PHY group appeared even more compromised presenting typical matrix swelling and poorly organized or absent cristae. On the contrary, mitochondria from mice supplemented with 500 mg/kg BW/day of ubiquinol alone (QH_2_) or in association with physical exercise (QH_2_ + PHY) appear slightly smaller but with well-preserved cristae. Mitochondrial membrane potential analysis (Figures 5(b) and 5(c)) confirmed that the altered mitochondrial ultrastructure observed in the PHY group was associated with significantly increased mitochondrial depolarization (+20% cells with depolarized mitochondria *vs.* SED group, *p* = 0.03, Figure 5(c)), suggesting that exercise might account for a bioenergetics impairment in aged muscles of 7-month-old SAMP8 mice. Notably, this increase was significantly counteracted following ubiquinol supplementation in association with regular physical exercise (QH_2_ + PHY) (−12.7%, *p* = 0.01), while QH_2_ alone was not able to decrease the basal level of depolarized cells which was similar to sedentary mice. These data suggest that ubiquinol supplementation in combination with regular physical exercise prevents exercise-dependent mitochondrial dysfunctions.

### 4.4. Combination of Physical Exercise and Ubiquinol Promotes Mitochondrial Biogenesis in the Muscles of the QH_2_ + PHY Group

**Figure 6:**
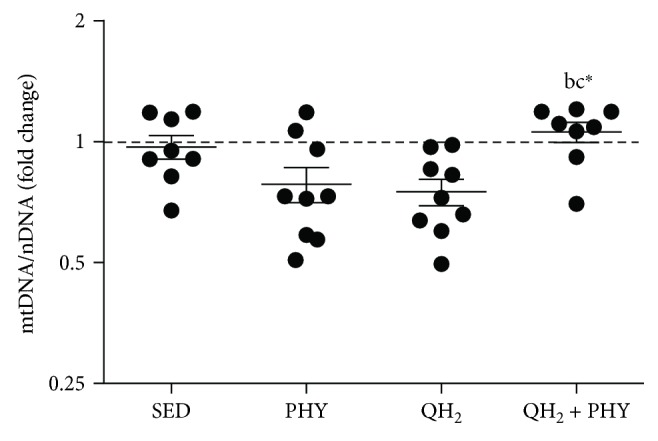
Fold change of copy number of mitochondrial DNA/nuclear DNA (mtDNA/nDNA) measured on *gastrocnemius* muscle in sedentary (SED), physical exercise (PHY), ubiquinol (QH_2_), and ubiquinol associated with physical exercise (QH_2_ + PHY) mouse groups (*n* = 10). ^∗^ *p* < 0.05; (b) = *vs.* PHY and c = *vs.* QH_2_.

**Figure 7:**
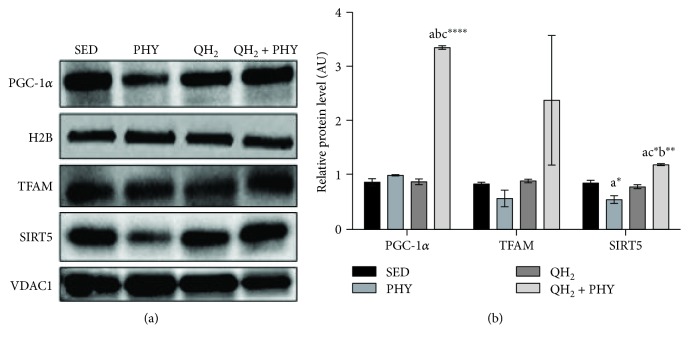
Western blot analysis (a) and relative protein quantification (b) of PGC-1*α*, TFAM, and SIRT5 expressed in tibialis anterior (TA) muscle, in sedentary (SED), physical exercise (PHY), ubiquinol (QH_2_), and ubiquinol associated with physical exercise (QH_2_ + PHY) mouse groups (*n* = 5). PGC-1*α* protein levels were normalized to H2B levels. TFAM and SIRT5 were normalized to VDAC1 levels. AU: arbitrary units. ^∗^ *p* < 0.05, ^∗∗^ *p* < 0.01, and ^∗∗∗∗^ *p* < 0.0001; (a) = *vs.* SED, (b) = *vs.* PHY, and (c) = *vs.* QH_2_.

Mitochondrial DNA content and PGC-1*α*, Tfam, and SIRT5 protein levels of TA muscles were analyzed to evaluate the mitochondrial biogenesis. Notably, QH_2_ + PHY treatment was not only able to preserve mitochondrial morphology and functionality (Figure 5) but also capable of modulating mitochondrial biogenesis. In particular, a significantly higher mtDNA copy number was detected in the QH_2_ + PHY group compared to the two treatments alone (QH_2_ + PHY *vs.* PHY, 1.35-fold change, *p* = 0.03, and QH_2_ + PHY *vs.* QH_2_, 1.41-fold change, *p* = 0.01, Figure 6). Moreover, the combined treatment promoted a highly significant increase in the expression of proteins involved in mitochondrial biogenesis, such as PGC-1*α* (+284.9%, *p* < 0.0001) and SIRT5 (+39.5%, *p* = 0.02), compared to sedentary mice (Figure 7). Regular physical exercise in association with ubiquinol supplementation was also able to increase the expression levels of TFAM although not in a significant manner. On the contrary, individual treatments, both physical exercise and ubiquinol supplementation, did not induce any changes in these markers, with the exception of a significant downregulation of SIRT5 in the trained mice.

### 4.5. Combination of Ubiquinol and Physical Exercise Thwarts Activation of Autophagy/Mitophagy Signals and Lowers Caspase 3-Dependent Apoptosis in the Muscles of QH_2_ + PHY Mice

**Figure 8:**
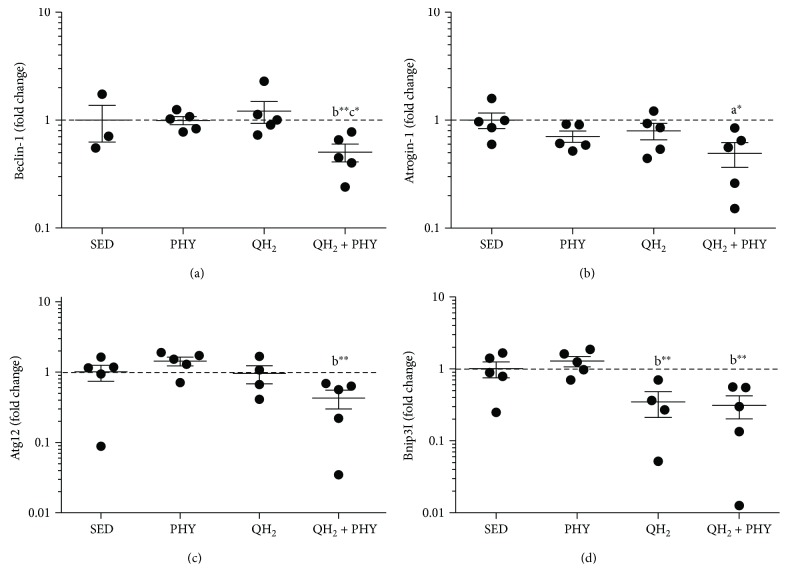
Gene expression (mRNA) expressed as fold change of genes Beclin (a), Atrogin (b), Atg12, (c) and Bnip3l (d) measured on *gastrocnemius* muscle in sedentary (SED), physical exercise (PHY), ubiquinol (QH_2_), and ubiquinol associated with physical exercise (QH_2_ + PHY) mouse groups (*n* = 5). ^∗^ *p* < 0.05 and ^∗∗^ *p* < 0.01; (a) = *vs.* SED, (b) = *vs.* PHY, and (c) = *vs.* QH_2_.

**Figure 9:**
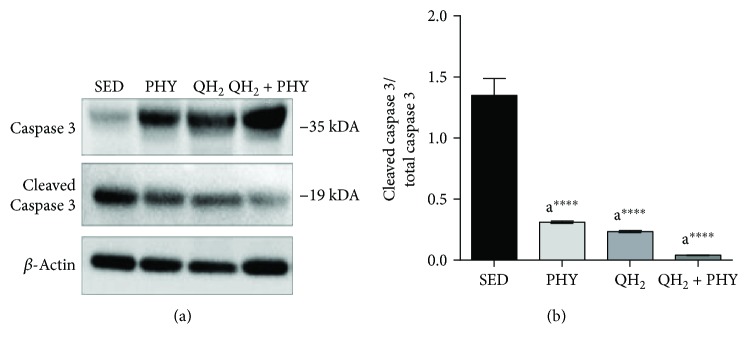
Immunoblot image (a) and relative protein quantification normalized to *β*-actin (b) of caspase 3 and cleaved caspase 3, measured on *tibialis anterior* muscle in sedentary (SED), physical exercise (PHY), ubiquinol (QH_2_), and ubiquinol associated with physical exercise (QH_2_ + PHY) mouse groups (*n* = 5). ^∗∗∗∗^ *p* < 0.0001; (a) = *vs.* SED.

To assess whether the different treatments impacted muscular autophagy/mitophagy, we analyzed the mRNA expression (Figure 8) of Beclin-1 (a), Atrogin-1 (b), Atg12 (c), and Bnip3l (d) genes encoding key players involved in both these degradation processes. The association of ubiquinol supplementation and physical exercise produced a significant decrease in the mRNA expression of Beclin-1 (−1.96-fold, *p* = 0.005), Atg12 (−3.34-fold, *p* = 0.003), and Bnip3l (−4.1-fold, *p* = 0.004) if compared to the PHY group. Atrogin-1 expression decreased significantly only compared to sedentary mice (*p* = 0.04). Ubiquinol supplementation alone was able to induce a significant decrease only for Bnip3l mRNA expression to a similar extent to the combined treatment of ubiquinol and physical exercise (−3.7-fold, *p* = 0.009). To determine whether apoptosis was also modulated, cleaved caspase 3 level was also examined via Western blot assay (Figure 9). Notably, despite that all the treatments were able to significantly decrease caspase 3-dependent apoptosis with respect to SED controls, QH_2_ + PHY combination triggered the most pronounced antiapoptotic effect in PHY (−76.9%), QH_2_ (−82.6%), and QH_2_ + PHY (−96.9%) mouse groups, respectively, compared to SED (*p* < 0.0001, Figures 9(a) and 9(b)). Overall, these data suggest that QH_2_ + PHY combination successfully lowers the expression of autophagy/mitophagy-associated genes and prevents apoptotic cell death inside the aging muscles.

## 5. Discussion

In the present study, senescence-accelerated prone 8 (SAMP8) mice, characterized by premature aging and high degree of oxidative stress [68], were used to investigate if a combined approach of mild physical exercise and ubiquinol (CoQH_2_) supplementation was able to improve mitochondrial function and preserve skeletal muscle health during aging. In our experimental settings, SAMP8 mice were treated with ubiquinol, physical exercise, and a combination of both for two months starting in the presarcopenia phase (5 months) until sarcopenia onset (7 months) [53]. As expected, the skeletal muscle of 7-month-old SED mice (used as control) presented high oxidative stress, damaged mitochondria, high extent of apoptosis, and mitophagy. While ubiquinol or physical exercise alone was able only to partially rescue these impairments, the combination of ubiquinol and physical exercise significantly improved the overall structural and functional status of the skeletal muscle. Skeletal muscle senescence is associated with decreased muscle mass and mitochondrial dysfunction, and the excessive production of mitochondrial ROS seems to strongly associate with the disruption of mitochondrial energy metabolism [19]. In this context, physical exercise has been proposed as a strategy to stimulate mitochondrial respiration and biogenesis counteracting muscle decline in older subjects [27, 28]. Nonetheless, some studies have shown that ROS production could indeed exacerbate the oxidative stress in senescent muscle, which is characterized by a severely impaired antioxidant response [32–34, 69]. For these reasons, the association of mild regular physical activity and antioxidant therapies could be a powerful strategy to minimize the adverse effects of exercise during aging. In particular, coenzyme Q in its reduced and active form (ubiquinol), being a key player both in the mitochondrial electron transport chain and in the antioxidant response in biological membranes [48], may represent an ideal candidate in improving oxidative status and functionality of the senescent muscle.

CoQ_10_ level correlates to high rates of metabolism, and for this reason, it is highest in organs such as the heart, kidney, and liver (114, 66.5, and 54.9 g/g tissue, respectively) [70], probably due to the large amounts of mitochondria where it is acting as an energy transfer molecule. In fact, coenzyme Q was first isolated from beef heart mitochondria, in 1957 [71].

Accordingly, at 7 months of age, skeletal muscle content of endogenous CoQ_9_ was significantly lower and more oxidized in comparison to cardiac muscle. Oral ubiquinol supplementation (500 mg/kg body weight/day) alone was unable to increase skeletal and cardiac muscle CoQ content and only the association of ubiquinol supplementation and mild treadmill running significantly increased the amount of both coenzymes in the cardiac muscle but not in the skeletal muscle. Increase of both coenzymes (endogenous CoQ_9_ and dietary absorbed CoQ_10_) in the heart suggests a higher biosynthesis rate that could be related to different mitochondrial requirements triggering both biosynthesis and incorporation. In the skeletal muscle, these changes that could be required for efficient tissue incorporation seem to occur at a much lower extent or might be less evident due to a lower mitochondrial content. Accordingly, Ernster and Dallner have previously shown that feeding rats with a comparable dosage of oxidized CoQ_10_ significantly increased its plasma content, while tissue CoQ_10_ accumulation was very moderate and variable in different tissues/organs [47]. In particular, skeletal muscle seems to have a very low ability to incorporate CoQ. However, Sohal and Forster showed that CoQ_10_ dietary supplementation in rodents was able to change the subcellular localization of CoQ, increasing the mitochondrial content of both coenzymes in various mitochondria-rich tissues, such as liver, heart, and skeletal muscle [72]. In another study, the same authors confirmed that skeletal muscle increase in CoQ_10_ following supplementation was the lowest in all analyzed tissues [73]. In the present study, we verified that the use of orally administered reduced CoQ_10_ did not provide any significant improvement in tissue uptake, showing results in line with previous reports where ubiquinone was used as active substance. This is a relevant observation since ubiquinol has been proposed as a more bioavailable form of Coenzyme Q_10_; nonetheless, in the proposed experimental condition, the oxidative state of ubiquinol does not seem to provide any significant improvement in terms of tissue uptake.

Taken together, these data suggest that ubiquinol dietary supplementation alone might not be enough to produce its cellular accumulation, but additional stimuli, such as physical activity and mitochondrial biogenesis, could improve ubiquinol incorporation [74]. Indeed, we reported that physical exercise could therefore act as a trigger for CoQ accumulation or rearrangement at the subcellular level. This effect was particularly evident in mitochondria-rich cardiac muscle, resulting in a significant increase in the overall cellular content. Moreover, even if we did not observe a significant CoQ accumulation in the skeletal muscle, we observed functional modifications at mitochondrial and cellular levels suggesting CoQ activity without accumulation in this tissue.

We focused our investigation on skeletal muscle considering its primary involvement in physical exercise. In the SAMP8 model, physical exercise alone produces heterogeneous responses at the cellular level. On the one hand, it had a clear ergogenic effect being able to promote an increase in skeletal muscle fiber size and to improve the oxidative status of endogenous coenzyme Q_9_. Accordingly, observational and intervention studies have demonstrated that physical exercise has a positive effect on muscle mass, muscle strength, and physical function in the older population [1, 2, 75, 76]. However, physical exercise also induced mitochondrial disturbances in terms of membrane depolarization. This result could be due to a reduced antioxidant activity [68] and to an intrinsic impairment of the electron transport chain [77]. Both conditions characterizing the presenescent SAMP8 mice might be further exacerbated by physical exercise in our experimental settings.

Notably, the combined treatment was able to counteract the mitochondrial impairment induced by physical exercise alone and also increased the mitochondrial number assessed as mtDNA/nDNA ratio. These data are confirmed by the analysis of PGC-1*α* muscle protein level, a key protein involved in the control of mitochondrial biogenesis, oxidative metabolism, and autophagy [23, 78, 79] which was significantly increased after 2 months of regular physical exercise associated with ubiquinol supplementation. Several studies reported that induction of PGC-1*α*, NRF-1, and Tfam expression [80–82] during physical exercise is triggered by oxidative stimuli [83]. On the contrary, aging-derived oxidative stress does not produce similar effects altering PGC-1*α* expression through different mechanisms [63].

Moreover, in our experimental model, increased fiber diameter in trained animals was not associated with a parallel increase in mtDNA copy number after 2 months of physical exercise, confirming that muscle hypertrophy was not linked with enhanced mitochondrial biogenesis. Shrinkage of the mitochondrial pool is a feature typical of the senescence process, and it is characterized by decreased enzymatic activity and level of mitochondrial proteins [21, 84, 85] as well as low mtDNA content [63, 86]. In this regard, it is remarkable that PGC-1*α* upregulation was associated with a concomitant increase in mtDNA copy number only in skeletal muscle of QH_2_ + PHY mice and not in mice subjected to single interventions.

Moreover, SIRT5, which has been recently found to protect mitochondria from fragmentation and degradation, by supporting mitochondrial elongation [87], was significantly increased following 2 months of ubiquinol supplementation and regular physical exercise, further supporting a positive effect of the QH_2_ + PHY combined treatment that could suggest improvement in mitochondria biogenesis.

Intriguingly, despite the improvements in the mitochondrial pool and the functionality observed in the QH_2_ + PHY group, an increase in muscle fiber size was produced by physical exercise alone and to a lower extent by the combination treatment. Indeed, muscle mass depends on different factors other than mitochondrial biogenesis, such as the balance between protein synthesis and degradation. van Wessel et al. described how high oxidative fibers are small in size, despite their high capacity for protein synthesis if compared to low oxidative fibers [88]. The authors suggest that cellular energy status may be crucial in mediating either a low oxidative phenotype and a large size or a high oxidative phenotype but a small size. They also suggest that oxidative fibers have a higher rate of muscle protein degradation in the presence of low cellular energy or high oxidative stress status, a condition similar to our experimental model.

During aging, senescent cells respond to a wide range of damaging stimuli produced by the accumulation of dysfunctional proteins and organelles, among which mitochondria play a pivotal role due to their bivalent role as source, target of ROS, and master regulator of programmed cell death processes. Throughout evolution, cells developed strategies like autophagy, mitophagy, and apoptosis to manage these constraints. These tightly regulated and interconnected processes are of pivotal importance also in the maintenance muscle homeostasis, which is dysregulated during aging and sarcopenia [89, 90]. In this context, ROS originating from mitochondria have been reported to activate both autophagy machinery [91] and caspase-dependent apoptosis [92].

In this regard, the combined treatment with ubiquinol and physical exercise not only ameliorated muscle oxidative stress and bioenergetics but also associated with a decreased expression of autophagy/mitophagy-associated genes, such as Bnip3l, Atg12, and Beclin-1 known to promote mitochondrial fragmentation and mitophagy and autophagosome formation [93, 94].

Finally, the combined treatment was also able to prevent caspase 3-dependent apoptosis. Since caspase cleavage cascade is a readout of mitochondria-associated apoptotic cell death [95–97], these data strengthen the positive impact of QH_2_ + PHY combination on mitochondria. Furthermore, previous studies have shown that cytochrome c release from mitochondria activates caspase 3 that in turn cleaves respiratory complex proteins exacerbating mitochondrial dysfunction increasing ROS production [92]. The loss of muscle mass, caused by an imbalance between muscle protein synthesis (MPS) and muscle protein breakdown (MPB) [98] and consequently decline in strength, can also be due to increased activity of the ubiquitin proteasome pathway (UPP), which is also responsible for mitochondrial protein quality control. Our data, showing a decrease in Atrogin-1 expression and myogenin (MyoG) protein level (supplementary material (available here)) and highlighting a potential effect of the combined treatment, suggest also a possible downregulation of UPP proteolytic pathway, which might concur to muscle health preservation [99].

Taking into consideration the limitations of this study, associated to the fact that we did not take into account muscle fiber type composition and we did not measure the actual intramuscular ROS levels, overall in this context, our data demonstrate that ubiquinol might directly prevent cell death by acting both as mitochondrial nutrient and as ROS scavenger.

## 6. Conclusion

In conclusion, the present study shows that ubiquinol supplementation and physical exercise synergize at improving mitochondrial functionality, counteracting the deleterious effects of physical exercise-induced ROS in the muscles of a SAMP8 mouse model. These results suggest that ubiquinol could be a powerful dietary supplement in sports nutrition and in particular in the elderly. The use of antioxidants in sports practice is still a debated topic, because it was demonstrated that some of these molecules are able to turn off the hormetic signals generated by physical exercise. However, antioxidant compounds represent a very heterogeneous family of molecules with different targets and cellular tropism so that evidences reported for some of them should not be simply extended to all molecules with similar activities. Moreover, data available in the scientific literature referring to a quenching effect of antioxidants on adaptive response commonly refer to trained healthy subjects. Our study shows that ubiquinol, while reducing harmful effects generated by physical exercise, improves exercise-induced hormetic response in a model characterized by elevated oxidative stress and prone to premature aging such as SAMP8 mice.

## Figures and Tables

**Table 1:** pRT-PCR primers for nDNA and mtDNA.

Target	Primer sequence_forward	Primer sequence_reverse
36B4	5′-CGACCTGGAAGTCCAACTAC-3′	5′-ATCTGCTGCATCTGCTTG-3′
COX1	5′-TCTACTATTCGGAGCCTGAGC-3′	5′-CAAAAGCATGGGCAGTTACG-3′

**Table 2:** Summary of used antibodies.

Primary antibodies			
Antigen	Antibody	Dilution	Brand

PGC-1*α*	Mouse monoclonal anti-PGC-1*α*	1 : 1000	Millipore
TFAM	Rabbit monoclonal anti-TFAM	1 : 2000	Abcam
VDAC1	Mouse monoclonal anti-VDAC1	1 : 1000	Abcam
H2B	Rabbit polyclonal anti-H2B	1 : 1000	Abcam
*β*-Actin	Mouse monoclonal anti-*β*-actin	1 : 1000	Cell Signaling Technology
Caspase 3	Rabbit polyclonal anti-caspase 3	1 : 1000	Cell Signaling Technology
Cleaved caspase 3	Rabbit polyclonal anti-cleaved caspase 3 (Asp175)	1 : 1000	Cell Signaling Technology
SIRT5	Rabbit monoclonal anti-SIRT5	1 : 1000	Cell Signaling Technology

Secondary antibodies			
Antibody		Dilution	Brand

HRP-conjugated goat anti-mouse IgG (H&L)		1 : 3000	Calbiochem
HRP-conjugated goat anti-rabbit IgG (H&L)		1 : 20000	Sigma-Aldrich

**Table 3:** Summary of used primers for Beclin, Atg12, Bnip3l, Atrogin, and GAPDH.

Target	Primer sequence_forward	Primer sequence_reverse
Beclin	5′-TGAATGAGGATGACAGTGAGCA-3′	5′-CACCTGGTTCTCCACACTCTTG-3′
Atg12	5′-TCCGTGCCATCACATACACA-3′	5′-TAAGACTGCTGTGGGGCTGA-3′
Bnip3l	5′-TTGGGGCATTTTACTAACCTTG-3′	5′-TGCAGGTGACTGGTGGTACTAA-3′
Atrogin	5′-GCAAACACTGCCACATTCTCTC-3′	5′-CTTGAGGGGAAAGTGAGACG-3′
GAPDH	5′-TCAACGGCACAGTCAAGG-3′	5′-ACTCCACGACATACTCAGC-3′

## Data Availability

The data used to support the findings of this study are available from the corresponding author upon request.

## Supplementary Materials

Supplementary Materials Immunoblot image and relative protein quantification of myogenin (MyoG) normalized to H2B (Figure 10) were measured on *tibialis anterior* muscle of all mouse groups. The results showed how ubiquinol supplementation, in association with physical exercise (QH_2_ + PHY mouse group), was significantly (^∗^ *p* < 0.05) able to prevent the increase in MyoG protein level induced after physical exercise alone (PHY mouse group). These data highlight a protective role of ubiquinol towards the deleterious effect of mild physical exercise in an old skeletal muscle of senescence-accelerated mice, suggesting also a possible downregulation of the UPP proteolytic pathway. Figure 10: immunoblot image and relative protein quantification of myogenin (MyoG) normalized to H2B measured on *tibialis anterior* muscle in sedentary (SED), physical exercise (PHY), ubiquinol (QH_2_), and ubiquinol associated with physical exercise (QH_2_ + PHY) mouse groups (*n* = 5), ^∗^ *p* < 0.05.
