# Supplementary material for: Combination of Coenzyme Q10 Intake and Moderate Physical Activity Counteracts Mitochondrial Dysfunctions in a SAMP8 Mouse Model
Source: Oxid Med Cell Longev. 2018 Oct 24;2018:8936251. doi: 10.1155/2018/8936251 (PMC6220380; doi:10.1155/2018/8936251)
Supplement: Supplementary Materials — Immunoblot image and relative protein quantification of myogenin (MyoG) normalized to H2B (Figure 10) were measured on tibialis anterior muscle of all mouse groups. The results showed how ubiquinol supplementation, in association with physical exercise (QH2 + PHY mouse group), was significantly (∗ p < 0.05) able to prevent the increase in MyoG protein level induced after physical exercise alone (PHY mouse group). These data highlight a protective role of ubiquinol towards the deleterious effect of mild physical exercise in an old skeletal muscle of senescence-accelerated mice, suggesting also a possible downregulation of the UPP proteolytic pathway. Figure 10: immunoblot image and relative protein quantification of myogenin (MyoG) normalized to H2B measured on tibialis anterior muscle in sedentary (SED), physical exercise (PHY), ubiquinol (QH2), and ubiquinol associated with physical exercise (QH2 + PHY) mouse groups (n = 5), ∗ p < 0.05. [file 8936251.f1.pdf]

SUPPLEMENTARY MATERIALS

10

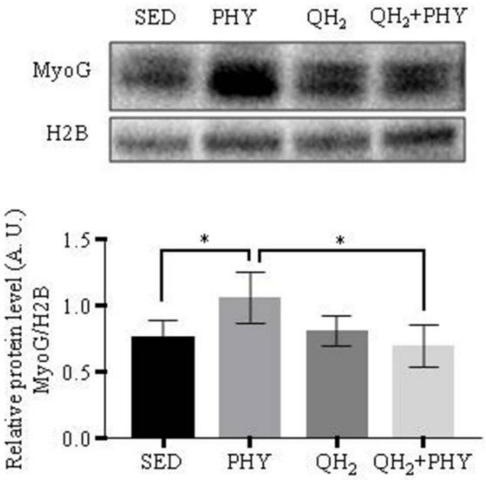

Figure 10. Immunoblot image and relative protein quantification normalized H2B of myogenin (MyoG), measured on *tibialis anterior* muscle in sedentary (SED), physical exercise (PHY), ubiquinol (QH_2) and ubiquinol associated with physical exercise (QH_2+PHY) mice groups (n=5). *p<0.05
